# Revealing cholesterol effects on PEGylated HSPC liposomes using AF4–MALS and simultaneous small- and wide-angle X-ray scattering

**DOI:** 10.1107/S1600576723005393

**Published:** 2023-07-25

**Authors:** Ting-Wei Hsu, Ching-Hsun Yang, Chun-Jen Su, Yin-Tzu Huang, Yi-Qi Yeh, Kuei-Fen Liao, Tien-Chang Lin, Orion Shih, Ming-Tao Lee, An-Chung Su, U-Ser Jeng

**Affiliations:** a National Synchrotron Radiation Research Center, 101 Hsin-Ann Road, Hsinchu Science Park, Hsinchu 300094, Taiwan; b Department of Chemical Engineering, National Tsing Hua University, Hsinchu 300044, Taiwan; c Department of Physics, National Central University, Zhongli 320317, Taiwan; d College of Semiconductor Research, National Tsing Hua University, Hsinchu 300044, Taiwan; University of Sydney, Australia

**Keywords:** drug-carrying liposomes, phospho­lipid membranes, cholesterol effects, asymmetric flow field-flow fractionation, multi-angle light scattering, AF4-MALS, small-angle X-ray scattering, wide-angle X-ray scattering, SAXS–WAXS

## Abstract

Liposome development is of great interest owing to increasing requirements for efficient drug carriers. The structural features and thermal stability of such liposomes are crucial in drug transport and delivery. Reported here are the results of the structural characterization of PEGylated liposomes via small- and wide-angle X-ray scattering and an asymmetric flow field-flow fractionation (AF4) system coupled with differential refractive-index detection, multi-angle light scattering (MALS) and dynamic light scattering. This integrated analysis of the exemplar PEGylated liposome formed from hydrogenated soy phosphatid­yl­choline (HSPC) with the addition of cholesterol reveals an average hydro­dynamic radius (*R*_h_) of 52 nm with 10% polydispersity, a comparable radius of gyration (*R*_g_) and a major liposome particle mass of 118 kDa. The local bilayer structure of the liposome is found to have asymmetric electronic density profiles in the inner and outer leaflets, sandwiched by two PEGylated outer layers *ca* 5 nm thick. Cholesterol was found to effectively intervene in lipid chain packing, resulting in the thickening of the liposome bilayer, an increase in the area per lipid and an increase in liposome size, especially in the fluid phase of the liposome. These cholesterol effects show signs of saturation at cholesterol concentrations above *ca* 1:5 cholesterol:lipid molar ratio.

## Abstract 2

Small- and wide-angle X-ray scattering and asymmetric flow field-flow fractionation integrated with multi-angle light scattering (AF4–MALS) are used to characterize PEGylated liposomes of hydrogenated soy phosphatidylcholine (HSPC) that have potential for drug delivery. Observed local and global structural changes of the phospho­lipid bilayers reveal the effects of cholesterol on PEGylated HSPC liposomes.

## 1. Introduction

Liposomes, often containing unilamellar vesicles of phospho­lipids, have seen increasing utilization as nanocarriers for drug delivery (Lombardo & Kiselev, 2022 ▸). In such applications, the physical and chemical stabilities of liposomes for traversing complex biological environments under different conditions, such as temperature and pH, are critical. Recent studies have shown that cholesterol can significantly increase the thermal stability and mechanical properties of polyethyl­ene glycol-coated (PEGylated) liposomes (Geisler *et al.*, 2020 ▸; Nakhaei *et al.*, 2021 ▸; Shoji *et al.*, 1998 ▸). The improved liposome performance in solution can be attributed to the intervention of cholesterol in the phospho­lipid chain packing (Faria *et al.*, 2019 ▸); however, how cholesterols intervene in phospho­lipids for nano-scaled segregation in vesicle bilayers remains to be elucidated. Such information would be of help in tuning the membrane fluidity and permeability of the liposomes, hence facilitating the liposome uptake or release of drug molecules (Faria *et al.*, 2019 ▸; Nakhaei *et al.*, 2021 ▸; Li *et al.*, 2019 ▸) in different environments.

For drug-carrying purposes, liposomes are often designed to have a large enclosed water core on the order of about 100 nm in diameter; to further improve the structural stability during drug transportation and delivery, cholesterol, sucrose and polyethyl­ene glycol lipids are mixed with phospho­lipids to form unilamellar vesicles of a core–multishell structure. Previously, small- and wide-angle X-ray (SAXS and WAXS) or neutron scattering were used to reveal the bilayer features (a few nanometres thick) and the phospho­lipid chain packing (Sreij *et al.*, 2019 ▸; Hirai *et al.*, 2013 ▸). However, the multi-component liposomes are subject to environmental stimulation during drug loading or release, leading to correlated local and global structural changes (Lorena *et al.*, 2012 ▸; Schilt *et al.*, 2016 ▸). Simultaneous observations of the global and local bilayer structural information of liposomes would be of help in understanding their drug-carrying and -delivery efficiency (Nakhaei *et al.*, 2021 ▸; Li *et al.*, 2019 ▸). In this study, we have integrated an asymmetric flow field-flow fractionation (AF4) system into multi-angle light scattering (MALS), dynamic light scattering (DLS) and differential refractive-index (dRI) spectrometers, to reveal the structural features of a model PEGylated liposome of hydrogenated soy phosphatidylcholine (HSPC). Together with simultaneous SAXS–WAXS, our combined analysis elucidates collective global and local structural changes of the HSPC liposome on incorporation of cholesterol, especially during the gel-to-fluid phase transition of the liposome.

## 2. Experimental

### 2.1. Sample preparation

The HSPC (l-α-phosphatidylcholine) liposome powder used consists of phospho­lipids of 1,2-dipalmitoyl-*sn*-glycero-3-phospho­choline (DPPC) and 1,2-distearoyl-*sn*-glycero-3-phospho­choline (DSPC) in the molar ratio 1:8. The powder was mixed with cholesterol, sucrose and PEGylated lipid 1,2-distearoyl-*sn*-glycero-3-phospho­ethano­lamine-*N*-[meth­oxy­(poly­ethyl­ene glycol)-2000] (mPEG2000-DSPE) with molar ratios HSPC:mPEG2000-DSPE:cholesterol = 9:1:*x* (with *x* > 4). The mixed sample powder was used in liposome solution preparation of 10 m*M* HSPC for AF4/MALS/DLS/dRI and SAXS–WAXS measurements (Dominik *et al.*, 2020 ▸). Sample solutions of 10 m*M* HPSC, without the addition of cholesterol, were also prepared with a similar molar composition of 1 m*M* DPPC:8.0 m*M* DSPC, and 1 m*M* mPEG2000-DSPE, without cholesterol, in co-extrusion processing as previously reported (Yang *et al.*, 2019 ▸; Mineart *et al.*, 2017 ▸).

### 2.2. AF4–MALS measurements

**Figure 1:**
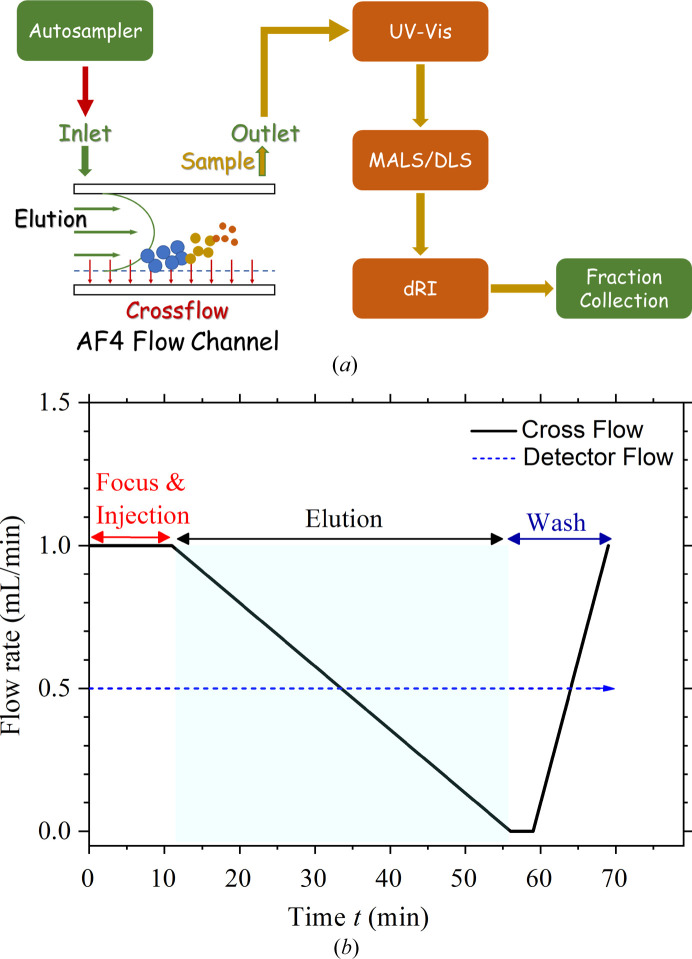
(*a*) Integrated AF4-MALS system, comprising an autosampler in the beginning and the AF4 of Wyatt Eclipse DualTec, followed by UV–vis absorption, MALS, DLS and dRI spectrometers, and terminated with a fraction collector. (*b*) Programmed elution-rate profile over the AF4-MALS elution (∼70 min) of the liposome sample solutions. The AF4 flow parameters are summarized in Table S1.

A Wyatt Eclipse DualTec system for AF4 was connected to a Wyatt-DAWN MALS spectrometer (with 18-angle light scattering detectors) and an Optilab dRI detector for determination of mass and radius of gyration *R*_g_; one of the 18 MALS detectors was replaced by a DLS device to determine the hydro­dynamic radius *R*_h_ of the liposomes (Fig. 1 ▸). Sample solutions of 2–30 µl were injected into the AF4 system and measured using a trapezoidal 265 mm-long channel of an RC 10 kDa cut-off membrane and a spacer for a channel height of 350 µm at 293 K. The AF4 parameters used are summarized in Table S1 of the supporting information. The refractive-index increment d*n*/d*c* = 0.146 ml g^−1^, used for deducing the mass of the PEGylated HSPC liposome, was determined from a separate measurement, with the integrated area of the elution profile of the sample solution with the AF4 channel path bypassed (to avoid loss of sample). Assuming 100% mass recovery, the *ASTRA* program (WYATT Technology) was employed to calculate the d*n*/d*c* value from the integrated elution peak area and prescribed sample weight. Details of AF4 analysis were reported previously for liposome characterization (Écija-Arenas *et al.*, 2021 ▸).

### 2.3. Small- and wide-angle X-ray scattering

SAXS and WAXS (SWAXS) measurements were performed at the 13 A BioSWAXS beamline of the Taiwan Photon Source at the National Synchrotron Radiation Research Center. The SWAXS data were collected with an X-ray beam energy of 15.0 keV (or wavelength λ = 0.8266 Å) using the two synchronized in-vacuum detectors Eiger X 9M (SAXS) and 1M (WAXS) of the beamline positioned at sample-to-detector distances of 2500 and 180 mm, respectively. The scattering vector magnitude *q* = 4πλ^−1^sinθ (with the scattering angle 2θ) and the projection angles of the WAXS detector plane were calibrated using a mixed powder of silver behenate and lanthanum hexaboride (LaB_6_). The absolute intensity (in cm^−1^) was calibrated using water scattering intensity (Shih *et al.*, 2022 ▸). The sample solutions were sealed in thermostated quartz capillaries (2 mm diameter and 20 µm wall thickness) and measured at 25, 40, 50 and 70°C. SAXS data were analyzed using the five-layer model of sharp scattering-length-density (SLD) interfaces, known as the core–multishell model, available in the *SASView* software platform (https://www.sasview.org/). The *X+* software with available Gaussian electron density profiles was also used in SAXS data analysis (Ben-Nun *et al.*, 2010 ▸).

## 3. Results and discussion

### 3.1. AF4-MALS results

**Figure 2:**
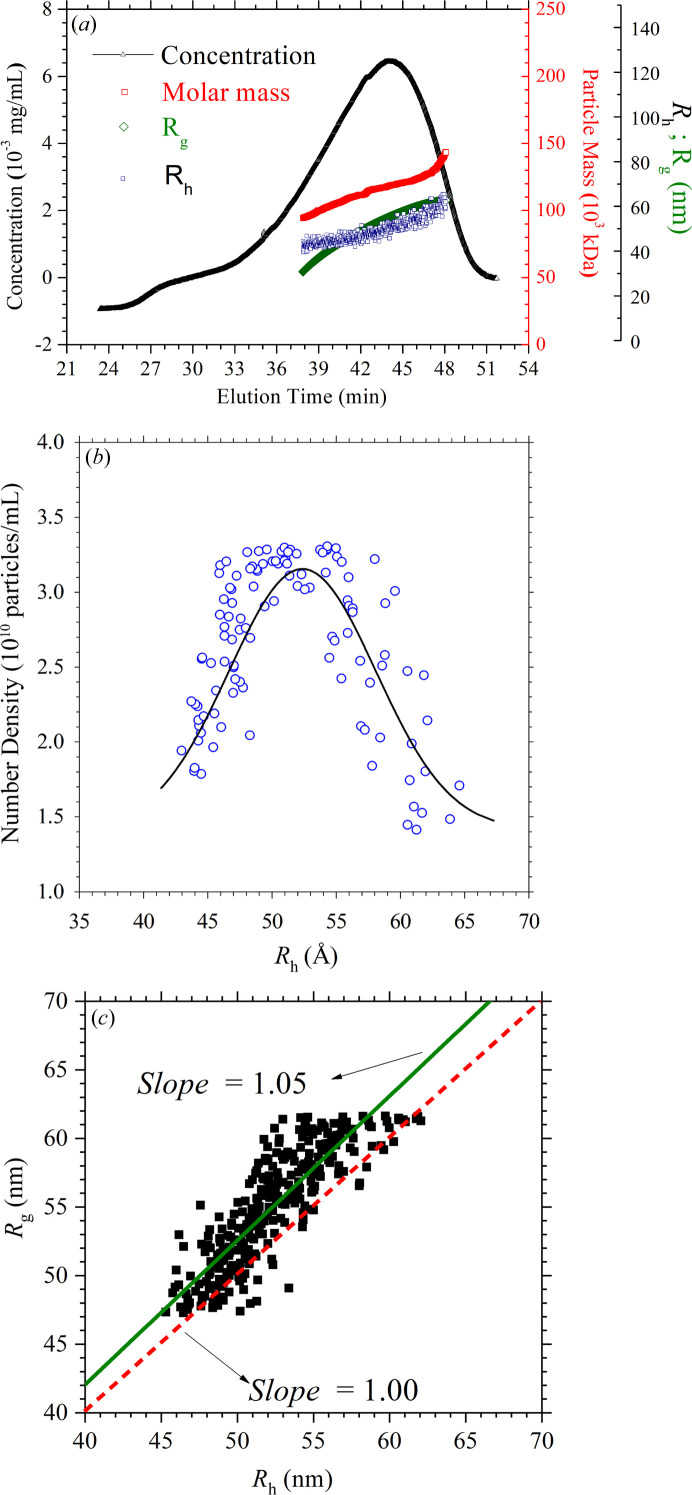
(*a*) Evolution of the liposome concentration, particle mass, *R*_g_ and *R*_h_ measured over the AF4 flow of the sample solution of PEGylated HSPC liposome with cholesterol, with *R*_g_ and *R*_h_ deduced from the MALS and DLS data, respectively. The mass was deduced from the combined analysis of MALS and dRI data. (*b*) Derived number-density distribution of the liposome as a function of *R*_h_, fitted with a Gaussian profile (solid curve). (*c*) *R*_g_ versus *R*_h_ presentation. Data are fitted with a solid line (slope *R*_g_/*R*_h_ = 1.05). Also shown is a red dashed line (slope = 1.0) representing the shape factor of ideal shells with *R*_g_/*R*_h_ = 1.

The AF4/MALS/DLS/dRI results for the HSPC liposomes with cholesterol are shown in Fig. 2 ▸(*a*), revealing the number-average particle mass *M*_n_ of 118 kDa, with *M*_w_/*M*_n_ = 1.0 (with weight-averaged mass *M*_w_). Also shown in Fig. 2 ▸(*a*) are the deduced hydro­dynamic radius *R*_h_ and radius of gyration *R*_g_ from the DLS and MALS–dRI data, respectively. Dividing the elution mass concentration by the particle mass deduced (Fig. 2 ▸) leads to the number density of the liposomes [Fig. 2 ▸(*b*)] as a function of *R*_h_; the result reveals a distribution peak at *R*_h_ = 52.4 nm and a polydispersity of *ca* 10% (Parot *et al.*, 2020 ▸). Fig. 2 ▸(*c*) presents the *R*_g_ versus *R*_h_ plot to illustrate the Burchard–Stockmayer shape factor *S*_f_ = *R*_g_/*R*_h_ (Mukherjee & Hackley, 2018 ▸) for the liposome. This falls close to the line of *S*_f_ = 1, corresponding to an ideal thin spherical shell structure. Nevertheless, the average *S*_f_ value (1.05) deduced is slightly above unity, which can be attributed to possible deformations of the liposome shape from a thin spherical shell under the asymmetric flow field of AF4. We note that from the known equation *R*_g_^2^ = (3/5)(*R*_1_^5^ − *R*_2_^5^)/(*R*_1_^3^ − *R*_2_^3^) for core–shell spheres (Feigin & Svergun, 1987 ▸), of core and shell radii *R*_1_ and *R*_2_, it can be deduced that *R*_g_ reduces to (3/5)^1/2^ *R*_h_ for solid spheres (*i.e. R*_2_ = 0) and *R*_g_ ≃ *R*_h_ for thin spherical shells with *R*_2_ ≃ *R*_1_; namely, the shape factor *R*_g_/*R*_h_ = (3/5)^1/2^ of solid spheres is smaller than that (≃ 1) of thin spherical shells. Further, it can be deduced that the values *R*_g_^2^ = 1/5(*a* ^2^ + 2*b* ^2^) of ellipsoids (with the semi-major and semi-minor axes *a* and *b*) of a common volume have a minimum with *a* = *b* for spheroids. Therefore, the measured shape *S*_f_ = 1.05 for the liposomes suggests possible deformation of the liposomes from the ideal spherical shape (*S*_f_ = 1).

### 3.2. Liposome membrane bilayer structures

**Figure 3:**
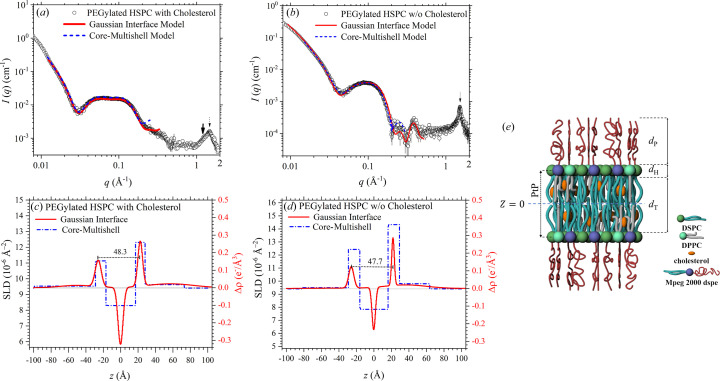
(*a*) SWAXS data of the PEGylated HSPC liposomes with cholesterol added. (*b*) Corresponding neat HPSC liposomes without cholesterol. The SAXS data are fitted using a five-layer core–multishell model (dash–dotted curves) with core radii of (*c*) 530 Å and (*d*) 428 Å and a multilayer model with five Gaussian electron density profiles (solid curves). In (*c*) and (*d*), the units of relative electron density Δρ (with respect to the water solvent) are used in the Gaussian interface model, with zero representing the absolute electron density of water (0.334 e^−^ Å^−3^). (*e*) Cartoon of the local structure of the PEGylated HSPC liposome, with *z* = 0 for the center of the bilayer. PtP represents the lipid head-to-head distance of the bilayer. The thin arrows in the WAXS region of (*a*) and (*b*) indicate the characteristic 2D hexagonal packing of the bilayer lipids of the liposomes.

Shown in Fig. 3 ▸(*a*) are the integrated SAXS–WAXS data of the PEGylated HSPC liposome with cholesterol added, revealing a characteristic broad hump centered around *q* ≃ 0.12 Å^−1^ from the typical vesical bilayers of *ca* 5 nm thickness. Also observed is an additional [compared with the SAXS data for the neat liposome without cholesterol; Fig. 3 ▸(*b*)] peak at *q* ≃ 0.05 Å^−1^ associated with the addition of cholesterol. Correspondingly, the broad hump centered around *q* ≃ 1.5 Å^−­1^ in the high-*q* region [Fig. 3 ▸(*a*)] indicates a significantly relaxed alkyl chain packing due to the intervening cholesterol. In contrast, the neat HSPC without cholesterol exhibits a relatively sharp peak at a similar *q* position, revealing a 2D hexagonal-like packing of the phospho­lipids with a Bragg *d* spacing of 4.2 Å (Geisler *et al.*, 2020 ▸; Sreij *et al.*, 2019 ▸).

To reveal the detailed bilayer structure of the liposomes, we fitted the SAXS data with a core–multishell model, having a five-layer SLD profile with sharp interfaces (Yang *et al.*, 2019 ▸; Mineart *et al.*, 2017 ▸); a multilayer model comprising five Gaussian electron density profiles for smooth density transitions across the sublayer interfaces (Schilt *et al.*, 2016 ▸; Ben-Nun *et al.*, 2010 ▸) is also used to fit the same sets of data. The five-layer core–multishell model comprises the central alkyl-dominated zone sandwiched by the head-group sublayers of the phospho­lipids, which are further sandwiched by two outer PEGylated layers, as illustrated in Fig. 3 ▸(*e*). As shown in Figs. 3 ▸(*a*) and 3 ▸(*b*), the SAXS data are better fitted in the higher-*q* region (>0.2 Å^−1^) using the asymmetric Gaussian electron density profiles compared with the core–multishell SLD profiles [Figs. 3 ▸(*c*) and 3 ▸(*d*)]; nevertheless, both models could fit the lower-*q* data equally well down to ∼0.01 Å^−1^, with qualitatively consistent electron density profiles. We note that the asymmetry in the electron density profile revealed consistently from both models is crucial in the data fitting. We also attempted a seven-layer core–multishell model fitting by adding an additional thin layer to the center of the lipid tail region; the fitting result, however, reduces to that of the five-layer model.

Figs. 3 ▸(*c*) and 3 ▸(*d*) illustrate the best-fitted asymmetric Gaussian electron density profiles for the HSPC liposome bilayer (Su *et al.*, 2013 ▸, 2018 ▸), which is sandwiched by two PEGylated layers each of *ca* 45 Å thickness. We attribute the higher electron density sublayers dominated by the phospho­lipid heads and the mPEG lipids in Figs. 3 ▸(*c*) and 3 ▸(*d*) to the inner leaflet of the bilayers. Presumably, the inner leaflet of the liposome bilayer, owing to its smaller shell radius (hence smaller shell area), might have tighter packing of the phospho­lipids and mPEG chains, resulting in sublayers of higher electron density. In contrast, the outer leaflet of a larger shell radius and facing open solvent tends to have more broadened peaks of lower electron density. On cholesterol intercalation, all the characteristic density peaks of the inner and outer leaflets of the bilayer [Fig. 3 ▸(*c*)] are broadened from that of the neat HSPC liposomes [Fig. 3 ▸(*d*)], leading to an enlarged bilayer thickness and peak-to-peak (PtP) distance (between the two phospho­lipid head sublayers of the inner and outer leaflets). Consistently, the thicker cholesterol-intercalated bilayer, with presumably larger bending modulus, is found to have larger liposome sizes as shown in Fig. S2 of the supporting information. These results suggest a significant association of the cholesterol with the alkyl chain zone. We note that the cholesterol–lipid interactions affect the global and local ordering of the bilayer concomitantly, as revealed from the nearly collapsed scattering hump at *q* ≃ 0.4 Å^−1^ (Su *et al.*, 2013 ▸) and the much broadened hump at *q* ≃ 1.5 Å^−1^ (the characteristic peak that represents the 2D hexagonal packing of the gel phase) from that observed for the neat HSPC liposome (Geisler *et al.*, 2020 ▸; Sreij *et al.*, 2019 ▸). Similar deteriorations in the scattering features are also consistently observed with the temperature-dependent SWAXS data of the pure PEGylated HSPC liposomes (Fig. S1), when the sample temperature increased from 25°C (gel phase) to 70°C (fluid phase).

### 3.3. Cholesterol effect on liposome chain packing revealed by WAXS analysis

**Figure 4:**
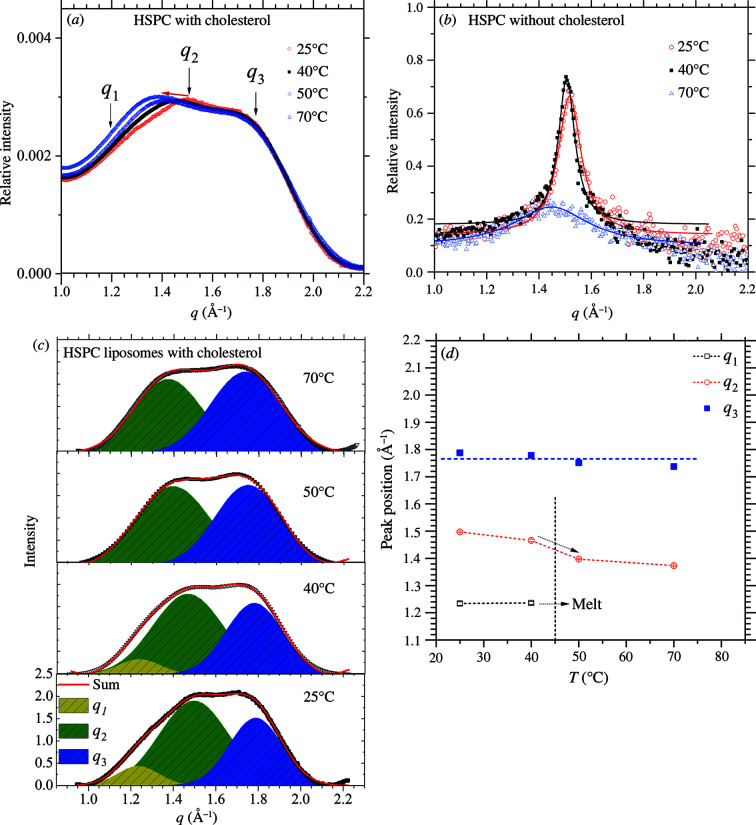
(*a*) Temperature-dependent WAXS data of the PEGylated HSPC liposome with the incorporation of cholesterol. (*b*) Parallel WAXS data for the PEGylated HPSC liposome without cholesterol. (*c*) Deconvoluted scattering humps from that shown in (*a*), with a common background subtracted. (*d*) Evolution of the three peak positions shown in (*c*), as indicated.

Neat HSPC liposomes were reported to have pre- and main gel-ordered-to-fluid-disordered phase transition temperatures at *T*_pre_ = 47.8°C and *T*_m_ = 53.6°C, respectively (Kitayama *et al.*, 2014 ▸). Shown in Fig. 4 ▸ are the WAXS data measured at 25, 40 and 70°C for the HSPC liposomes, with and without cholesterol. The neat PEGylated HSPC liposome manifests a primary sharp peak of the 2D hexagonal packing centered at *q*_2_ = 1.517 Å^−1^ at 25 and 40°C, which is significantly reduced at 70°C [Fig. 4 ▸(*b*)]. The corresponding coherent length *L*_c_ ≃ 2π/Δ*q*, deduced from the *q*_2_ peak width Δ*q*, increases from 117 Å at 25°C to 163 Å at 40°C, showing a pre- to main ordering behavior similar to that mentioned previously. At 70°C, the *q*_2_ peak deteriorates significantly for a much reduced *L*_c_ = 36 Å, illustrating the gel-to-fluid phase transition. From the *q*_2_ peak position, the deduced lipid–lipid *d* spacing *D* = 2π/*q*_2_ of the neat HSPC liposome changes from 4.14 to 4.17 to 4.34 Å, for 25, 40 and 70°C; the corresponding area per lipid *A*_L_ estimated from the 2D hexagonal packing with 16π^2^/( 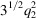 ) (Geisler *et al.*, 2020 ▸) increases from 39.6 to 40.2 to 43.5 Å^2^. The deduced feature sizes are summarized in Table 1 ▸.

In contrast, the WAXS data measured at 25°C for the HPSC liposome with cholesterol exhibit a convoluted broad hump that can be decomposed into three broad humps centered at *q*_1_ = 1.234 Å^−1^, *q*_2_ = 1.497 Å^−1^ and *q*_3_ = 1.788 Å^−1^; all three humps have similar small *L*_c_ values of 16–17 Å. We assign the *q*_2_ peak observed for the PEGylated HSPC liposomes with cholesterol to a deteriorated 2D hexagonal packing of the lipid–cholesterol complex. We notice that the *q*_2_ hump position of the liposome with cholesterol shifts to lower *q* values as the temperature increases from 25 to 40 to 50 to 70°C. The values deduced for *A*_L_ with cholesterol *A*_L-chol_ are also larger than those of the corresponding liposome without cholesterol, as shown in Table 1 ▸, especially in the fluid phase. The result suggests that cholesterol can interact substantially with the lipid chains, especially in the fluid phase of reduced lipid self-interactions.

We notice that *q*_1_ and *q*_3_ show little or no peak position shifting following the temperature changes, and the *q*_1_ peak even disappears at 50°C [Fig. 4 ▸(*a*)]. It is likely that these two peaks are associated with the packing of excess cholesterol phase segregated from the 2D hexagonal domains of the lipid–cholesterol complex, or an additional orthorhombic-like packing as suggested in previous reports (Sreij *et al.*, 2019 ▸; Geisler *et al.*, 2020 ▸). To further clarify the origin of these two peaks, we measured cholesterol-concentration-dependent SWAXS (Fig. S3). The results indeed show that the *q*_1_ and *q*_3_ peaks emerge with higher cholesterol content roughly above the molar ratio HSPC:mPEG:cholesterol = 9:1:2 (*i.e.* 20% cholesterol). The *q*_1_ peak of lower thermal stability may be associated with the 2D monolayer packing of cholesterol (Rapaport *et al.*, 2001 ▸); the less temperature-dependent *q*_3_ peak, however, may be of different origin.

## 4. Conclusions

SAXS–WAXS and AF4 coupled with MALS, DLS and dRI are used to successfully determine the mass, size and bilayer structural features of the PEGylated HSPC liposome. Cholesterol is found to significantly affect the lipid chain packing of the liposome, leading to thickening of the bilayer, an increase in *A*_L_ and an increase in the liposome size. These cholesterol effects show signs of saturation at higher cholesterol concentrations above *ca* 1:5 cholesterol:lipid molar ratio. Simultaneous SAXS–WAXS measurements correlate the concomitant structural changes in the inner and outer leaflets in the directions normal and parallel to the bilayer packing plane of the liposome upon intercalation of cholesterol and the gel-to-fluid phase transition.

## Supplementary Material

Supporting figures and tables. DOI: 10.1107/S1600576723005393/tj5034sup1.pdf

## Figures and Tables

**Table 1:** Summary of the peak-fitting parameters from the WAXS data shown in Fig. 4 ▸, including the peak center *q*_*i* = 1,2,3_, corresponding *d* spacing *D*_*i*_ and correlation length *L*_c_ (estimated from the peak width) *A*_L-chol_ and *A*_L_ are the areas per lipid deduced from the *q*_2_ peak position for the HSPC liposome bilayers with and without cholesterol, respectively; *ΔA* is the increment percentage of *A*_L-chol_ with respect to *A*_L_ at the corresponding temperature.

T (°C)	*q*_1_ (Å^−1^)/*D*_1_ (Å)/*L*_c_ (Å)	*q*_2_ (Å^−1^)/*D*_2_ (Å)/*L*_c_ (Å)	*q*_3_ (Å^−1^)/*D*_3_ (Å)/*L*_c_ (Å)	*A*_L-chol_ or *A*_L_ (Å^2^)	Δ*A* (%)
PEGylated HSPC liposome with cholesterol					
25	1.234/5.09/27	1.497/4.20/16	1.788/3.51/21	40.7	3
40	1.237/5.08/27	1.466/4.29/16	1.778/3.53/17	42.4	6
50	Melt	1.397/4.50/16	1.751/3.59/17	46.7	–
70	Melt	1.372/4.58/17	1.738/3.59/16	48.4	11

PEGylated HSPC liposome without cholesterol					
25		1.517/4.14/117		39.6	
40		1.506/4.17/163		40.2	
70		1.447/4.34/36		43.5	

## References

[bb1] Ben-Nun, T., Ginsburg, A., Székely, P. & Raviv, U. (2010). *J. Appl. Cryst.* **43**, 1522–1531.

[bb2] Dominik, D., Grzegorz, C., Sebastian, K. & Marek, L. (2020). *Langmuir*, **36**, 3826–3835.

[bb3] Écija-Arenas, Á., Román-Pizarro, V. & Fernández-Romero, J. M. (2021). *J. Chromatogr. A*, **1636**, 461798. 10.1016/j.chroma.2020.461798 33341435

[bb4] Faria, M. J., Machado, R., Ribeiro, A., Gonçalves, H., Real Oliveira, M. E. C. D., Viseu, T., das Neves, J. & Lúcio, M. (2019). *Pharmaceutics*, **11**, 485. 10.3390/pharmaceutics11090485 PMC6781289 31540519

[bb5] Feigin, L. A. & Svergun, D. I. (1987). *Structure Analysis by Small-Angle X-ray and Neutron Scattering* New York: Plenum Press.

[bb6] Geisler, R., Prévost, S., Dattani, R. & Hellweg, T. (2020). *Crystals*, **10**, 401.

[bb7] Hirai, M., Kimura, R., Takeuchi, K., Sugiyama, M., Kasahara, K., Ohta, N., Farago, B., Stadler, A. & Zaccai, G. (2013). *Eur. Phys. J. E*, **36**, 74. 10.1140/epje/i2013-13074-3 23852578

[bb9] Kitayama, H., Takechi, Y., Tamai, N., Matsuki, H., Yomota, C. & Saito, H. (2014). *Chem. Pharm. Bull.* **62**, 58–63. 10.1248/cpb.c13-00587 24390493

[bb11] Li, T., Clulow, A. J., Nowell, C. J., Hawley, A., Cipolla, D., Rades, T. & Boyd, B. J. (2019). *J. Colloid Interface Sci.* **555**, 361–372. 10.1016/j.jcis.2019.07.081 31398564

[bb12] Lombardo, D. & Kiselev, M. A. (2022). *Pharmaceutics*, **14**, 543. 10.3390/pharmaceutics14030543 PMC8955843 35335920

[bb13] Lorena, R.-M., Marina, I. G. & Fausto, S. (2012). *Langmuir*, **28**, 12851–12860.

[bb8] Mineart, K. P., Kelley, E. G., Nagao, M. & Prabhu, V. M. (2017). *Soft Matter*, **13**, 5228–5232. 10.1039/c7sm00960g PMC11112619 28730191

[bb14] Mukherjee, A. & Hackley, V. A. (2018). *Analyst*, **143**, 731–740. 10.1039/c7an01739a PMC6057617 29322138

[bb15] Nakhaei, P., Margiana, R., Bokov, D. O., Abdelbasset, W. K., Jadidi Kouhbanani, M. A., Varma, R. S., Marofi, F., Jarahian, M. & Beheshtkhoo, N. (2021). *Front. Bioeng. Biotechnol.* **9**, 705886. 10.3389/fbioe.2021.705886 PMC8459376 34568298

[bb17] Parot, J., Caputo, F., Mehn, D., Hackley, V. A. & Calzolai, L. (2020). *J. Controlled Release*, **320**, 495–510. 10.1016/j.jconrel.2020.01.049 PMC7146538 32004590

[bb18] Rapaport, H., Kuzmenko, I., Lafont, S., Kjaer, K., Howes, P. B., Als-Nielsen, J., Lahav, M. & Leiserowitz, L. (2001). *Biophys. J.* **81**, 2729–2736. 10.1016/S0006-3495(01)75915-2 PMC1301739 11606285

[bb19] Schilt, Y., Berman, T., Wei, X., Barenholz, Y. & Raviv, U. (2016). *Biochim. Biophys. Acta*, **1860**, 108–119. 10.1016/j.bbagen.2015.09.012 26391840

[bb20] Shih, O., Liao, K.-F., Yeh, Y.-Q., Su, C.-J., Wang, C.-A., Chang, J.-W., Wu, W.-R., Liang, C.-C., Lin, C.-Y., Lee, T.-H., Chang, C.-H., Chiang, L.-C., Chang, C.-F., Liu, D.-G., Lee, M.-H., Liu, C.-Y., Hsu, T.-W., Mansel, B., Ho, M.-C., Shu, C.-Y., Lee, F., Yen, E., Lin, T.-C. & Jeng, U. (2022). *J. Appl. Cryst.* **55**, 340–352. 10.1107/S1600576722001923 PMC8985603 35497659

[bb21] Shoji, K., Hayato, I., Takashi, H. & Hitoshi, M. (1998). *Biochim. Biophys. Acta*, **1374**, 1–8.

[bb22] Sreij, R., Dargel, C., Schweins, R., Prévost, S., Dattani, R. & Hellweg, T. (2019). *Sci. Rep.* **9**, 5542. 10.1038/s41598-019-41865-z PMC6447539 30944386

[bb23] Su, C.-J., Lee, M. T., Liao, K. F., Shih, O. & Jeng, U. S. (2018). *Phys. Chem. Chem. Phys.* **20**, 26830–26836. 10.1039/c8cp02861c 30137074

[bb24] Su, C.-J., Wu, S.-S., Jeng, U.-S., Lee, M.-T., Su, A. C., Liao, K. F., Lin, W. Y., Huang, Y. S. & Chen, C. Y. (2013). *Biochim. Biophys. Acta*, **1828**, 528–534. 10.1016/j.bbamem.2012.10.027 23123565

[bb25] Yang, C.-H., Lin, T.-L. & Jeng, U.-S. (2019). *Langmuir*, **35**, 9483–9492. 10.1021/acs.langmuir.9b00756 31287319

