# Supplementary material for: Revealing cholesterol effects on PEGylated HSPC liposomes using AF4–MALS and simultaneous small- and wide-angle X-ray scattering
Source: J Appl Crystallogr. 2023 Jul 25;56(Pt 4):988–93. doi: 10.1107/S1600576723005393 (PMC10405602; doi:10.1107/S1600576723005393)
Supplement: Supplementary file 1 [file j-56-00988-sup1.pdf]

JOURNAL OF APPLIED CRYSTALLOGRAPHY

Volume 56 (2023)

Supporting information for article:

Revealing cholesterol effects on PEGylated HSPC liposomes using AF4-MALS and simultaneous small- and wide-angle X-ray scattering

Ting-Wei Hsu, Ching-Hsun Yang, Chun-Jen Su, Yin-Tzu Huang, Yi-Qi Yeh, Kuei-Fen Liao, Tien-Chang Lin, Orion Shih, Ming-Tao Lee, An-Chung Su and U-Ser Jeng

1. Parameters used in the AF4 system

Table S1. Parameters used in the AF4 system for the liposome measurement. Note that the cross-flow was increased with a constant rate in the elution inject stage within 1 min, and subsequently stayed a constant flow rate in the elution stage.

Elution stage parameters	Duration [min]	Cross Flow Start [mL/min]	Cross Flow Stop [mL/min]
Elution	1.0	1.0	1.0
Focus	2.0	1.0	1.0
Focus inject	8.0	1.0	1.0
Elution	45.0	1.0	0.0
Elution inject	2.0	0.0	0.0
Elution	10.0	1.0	1.0

2. Temperature dependent SAXS-WAXS (SWAXS)

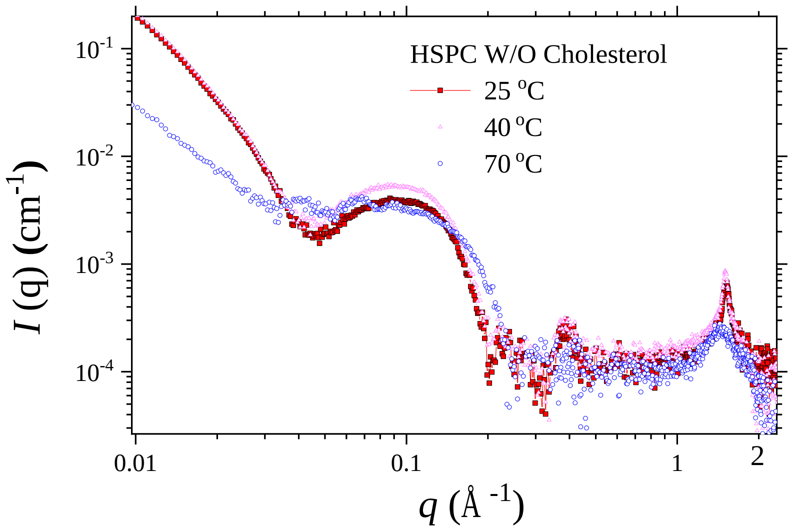

Figure S1. Temperature dependent SWAXS of the sample of HSPC without cholesterol. Note the concomitant melting of the peaks centered at q = 0.39 Å $^{-1}$ (correlated to the out-of-plane lipid bilayer packing) and q = 1.51Å $^{-1}$ (associated with the in-plane lipid packing within the bilayer) at 70 °C.

3. SWAXS for PEGylated HSPC liposomes of different cholesterol concentrations

3.1 Materials

Sample powders of 1,2-distearoyl-sn-glycero-3-phosphocholine (DSPC), 1,2-dipalmitoyl-sn-glycero-3-phosphocholine (DPPC), L-α-phosphatidylcholine, hydrogenated (Soy) (HSPC), 1,2-distearoyl-sn-glycero-3-phosphoethanolamine-N-[methoxy(polyethylene glycol)-2000] (ammonium salt) (mPEG2000-DSPE), and cholesterol (plant derived) were purchased from Avanti Polar Lipids (Alabaster, AL).

3.2. Sample solutions of the liposomes (unilamellar vesicles)

Prescribed l compositions of HSPC/mPEG2000-DSPE/Cholesterol, were dissolved in chloroform and shaken for uniformly mixing for molecular ratios of 9:1:0, 9:1:2, and 9:1:4. The sample solutions were dried at 55 °C controlled by water bath overnight and under vacuum for 30 min to remove residual solvent for sample films. Deionized water was added to the sample films for dispersion and shaken for 10 min. The solutions were then put into ultrasonic bath controlled at 60 °C for 50 min, and frozen and thawed between liquid nitrogen and 60 ℃ water for six times. The final suspension was extruded through polycarbonate filters of a pore size of 100 nm 10 times, respectively, for LUV. The diameter of the PEGylated HSPC liposomes, of different cholesterol concentrations, were estimated by dynamics light scattering (DLS, Malvern Zetasizer Nano S90) to be around 100 nm consequently. For SWAXS measurements, these sample solutions were respectively sealed in thermostated cells (2 mm dia. X-ray path length, sealed by two 8-μm Kapton films).

3.3 Data analysis

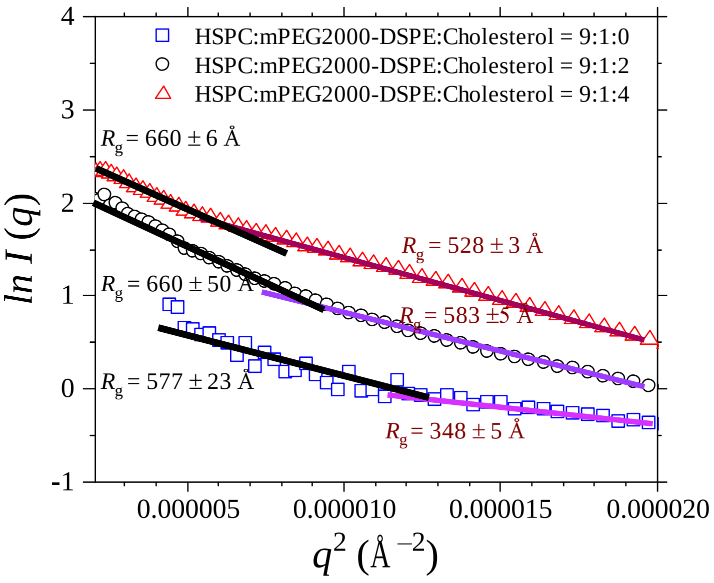

Figure S2. Guinier presentation for the SAXS data of the PEGylated HSPC liposomes (measured at 20 °C) with the molecular ratios of cholesterol indicated, showing a polydispersity feature (multi-slopes in the low-*q* region). Each set of data is fitted using the Guinier approximation (dotted lines) in the two *q*-ranges for the upper and lower bound of the R_g values of the size polydispersity. The liposomes in the two cases with cholesterol intercalated exhibit larger R_g sizes than that without.

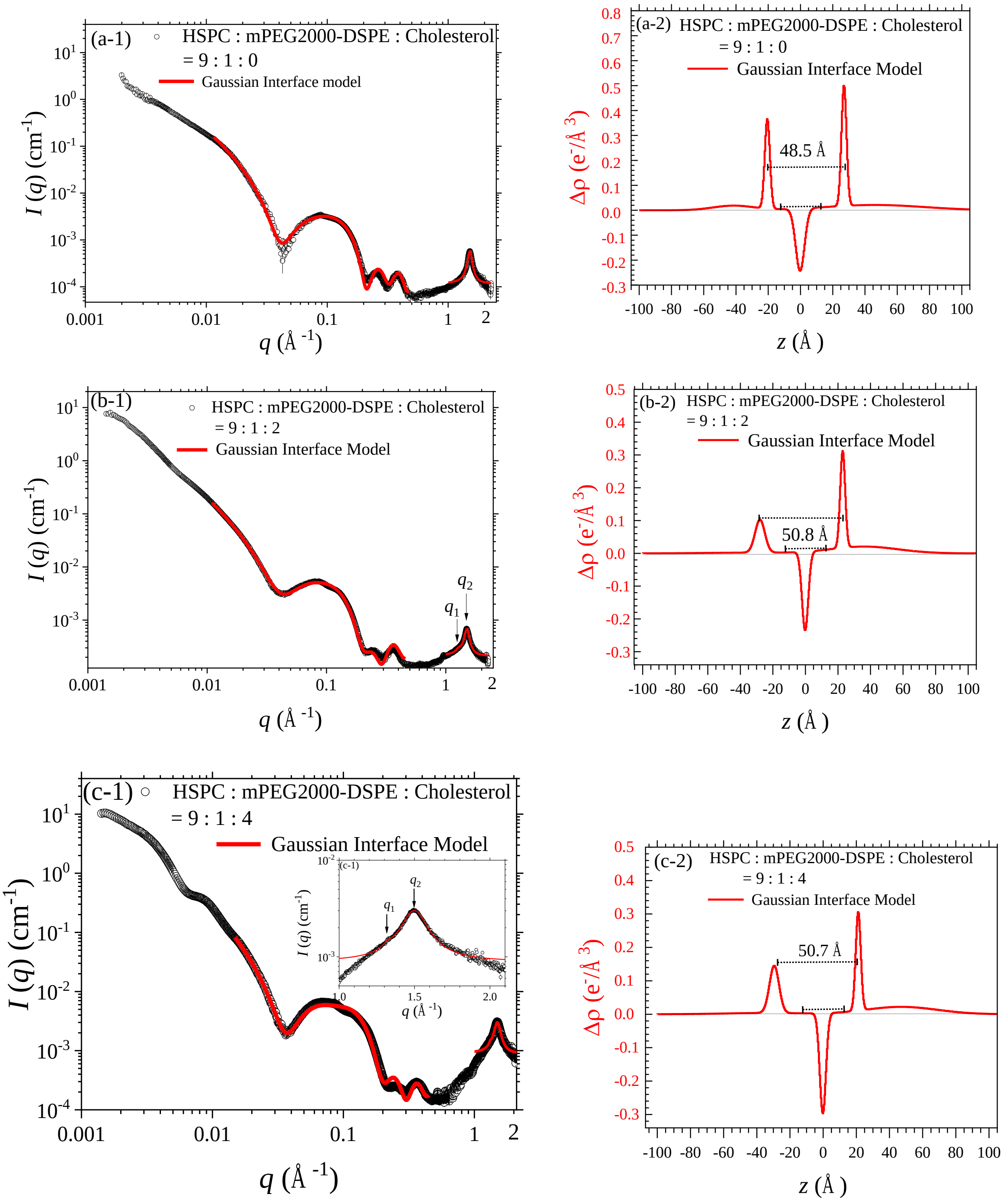

Figure S3. (a1-c1) SWAXS data for the PEGylated HSPC liposomes (measured at 20 °C) with different lipid-cholesterol molecular ratios indicated. The data are fitted with (a2-c2) the corresponding electron density profiles using the Gaussian interface model. The fitted parameters are summarized in Table S2. We note that the sample solutions were prepared with the HSPC powder received from the Co. Avanti Polar Lipids, whereas the sample solution for the data in Figure 3b (without cholesterol) was prepared from in-house mixing of DSPC : DPPC (1 : 8 molar ratio).

Table S2. The correspondingly fitted parameters using the Gaussian interface models of the SWAXS data (cf. Figure S3) measured at 20 °C for the PEGylated HSPC liposomes with different lipid-cholesterol molecular ratios, as indicated. Note that the units of relative electron density Δρ (with respect to the water solvent) are used in the Gaussian interface mode with the five Gaussian peaks of the peak width (full width at half maximum) *w*; Δρ = 0 represents the absolute electron density of water (0.334 e^-/Å ^{3}); *z* = 0 represents the center of the bilayer. PtP is the peak-to-peak distance of the inner and outer leaflets, as indicated in Figures S3. The values for the area per lipid $A_{L\text{-chol}}$ and A_L are the deduced area-per-lipid from the WAXS q_2 peak position (Figure S4) for the HSPC liposome bilayers with and without cholesterol.

HSPC : mPEG2000-DSPE : Cholesterol Molar Ratio	PtP (Å)	*z* (Å)	Δρ (e^-/Å $^{-3}$)	*w* (Å)	q_2 (Å $^{-1}$)	A_L, $A_{L\text{-chol}}$ (Å ^{2})
9 : 1 : 0	48.5	-41.39	0.01821	30.04	1.510	40.0
		-20.33	0.3596	3.71		
		0	-0.2500	6.42		
		27.21	0.4839	3.69		
		46.29	0.02120	73.03		
9 : 1 : 2	50.8	-35.54	0.002030	61.38	1.502	40.4
		-27.77	0.1004	7.01		
		0	-0.2400	4.55		
		23.07	0.2954	3.57		
		36.26	0.02061	48.72		
9 : 1 : 4	50.7	-35.01	0.003714	56.68	1.498	40.6
		-29.30	0.1415	6.75		
		0	-0.3000	4.34		
		21.35	0.2955	3.58		
		47.27	0.02210	50.77		

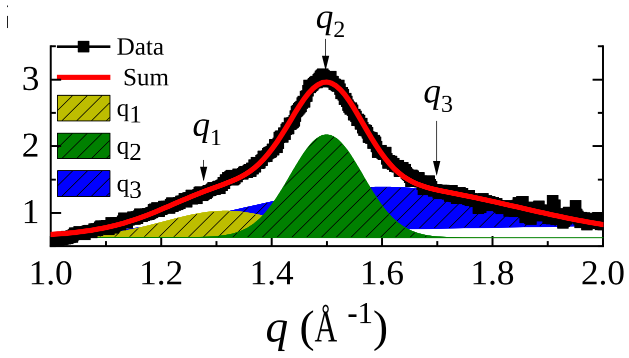

Figure S4. WAXS data for the PEGylated HSPC liposomes with the molar ratios of HSPC: mPEG2000-DSPE: Cholesterol 9:1:4. The data for (a) and (b) are fitted with a Gaussian peak centered at q_2 = 1.51 Å $^{-1}$, corresponding to the 2D hexagonal packing of the liposomes. (c) Data are fitted with three peaks with q_1 = 1.316 Å $^{-1}$, q_2 = 1.498 Å $^{-1}$ and q_3 ~ 1.70 Å $^{-1}$.
